# Transdermal Ketoprofen Versus Fentanyl Patches for Pain Management Post-cesarean Section: A Comparative, Randomized, Double-Blind Study

**DOI:** 10.7759/cureus.73635

**Published:** 2024-11-13

**Authors:** Sandip Baheti, Sharan Muruganantham

**Affiliations:** 1 Department of Anaesthesiology, Dr. D. Y. Patil Medical College, Hospital and Research Centre, Dr. D. Y. Patil Vidyapeeth (Deemed to be University), Pune, IND

**Keywords:** cesarean section, fentanyl patch, ketoprofen patch, postoperative analgesia, visual analogue scale (vas) for pain

## Abstract

Introduction: Postoperative pain management after a cesarean section is essential to promote mother-infant bonding and ease of breastfeeding. Transdermal patches present a viable alternative to oral medications, offering controlled drug delivery and better bioavailability while avoiding first-pass metabolism, all of which can facilitate smoother recovery and rehabilitation.

Methods: This comparative, randomized, double-blind study was conducted on 70 parturients scheduled for cesarean section under spinal anesthesia, classified as ASA II. Patients were randomly divided into two groups: Group K (transdermal ketoprofen patch, 30 mg) and Group F (transdermal fentanyl patch, 25 mcg). The patches were applied immediately after spinal anesthesia. Pain was assessed using the Visual Analogue Scale (VAS), and sedation was assessed using the Richmond Agitation-Sedation Scale for 24 hours.

Results: There was no statistically significant difference in the VAS scores between the two groups from two to 14 hours postoperatively. However, a statistically significant difference was observed at 16 hours (Group K 2 ± 0.87 vs. Group F 2.5 ± 0.86, p = 0.029) and 20 hours (Group K 1.77 ± 0.86 vs. Group F 2.33 ± 0.8, p = 0.011). Rescue analgesia was required in seven (20%) of the patients in Group K and 12 (34.29%) of the patients in Group F, though this difference was not statistically significant. At 20 hours, Group K had more alert and calm patients (33 (94.29%) vs. 26 (74.29%)), with fewer drowsy or restless patients compared to Group F (p = 0.044). At 24 hours, Group K had significantly fewer drowsy patients (0 (0%) vs. 13 (37.14%)) and more alert patients (34 (97.14%) vs. 19 (54.29%)), with fewer restless patients (p < 0.0001).

Conclusion: Both the ketoprofen patch (30 mg) and the fentanyl patch (25 mcg) provided comparable analgesia for the first 12 hours. However, at 16 and 20 hours, the ketoprofen patch was more effective than the fentanyl patch in providing analgesia. More patients in the fentanyl group were drowsy after 16 hours, though normal sleep patterns may have contributed to this observation.

## Introduction

The birth of a child is one of the most precious and joyous moments in a woman’s life. Affectionate touch, breastfeeding, and intimacy provide emotional healing. However, pain after a cesarean section can be a major obstacle. Various modalities are available to treat this pain, but side effects such as sedation, nausea, vomiting, and constipation can hinder the bonding between the mother and the baby. Postoperative pain after a lower segment cesarean section (LSCS) presents a significant medical challenge. It is common and often undertreated, which can delay the patient’s recovery, discharge, and rehabilitation. Studies from high-income countries show that inadequately managed postoperative pain following a cesarean section is associated with a higher risk of chronic pain and post-traumatic stress syndrome [1]. Therefore, it is crucial for anesthesiologists to understand the causes of pain and manage it with various techniques that ensure good efficacy and minimal side effects for both the mother and the newborn.

Methods to reduce postoperative pain include epidural analgesia, wound infiltration with local anesthetics, transversus abdominis plane blocks, transdermal patches, oral non-steroidal anti-inflammatory drugs (NSAIDs), and opioids [2]. While epidural analgesia is the most effective method, in developing countries like India, it may not be feasible to provide it to all cesarean section patients due to risks such as maternal hypotension, bladder incontinence, spinal epidural hematoma, headache, and limitations in movement. Local infiltration only provides short-term analgesic effects, and regional nerve blocks require expert skills for effective results. Transdermal patches offer several advantages over oral routes, including better bioavailability by bypassing first-pass metabolism [3], painless application, and controlled drug delivery through the skin [4].

Ketoprofen, a leading NSAID, has recently become available in patch forms. In addition to being a COX inhibitor, it also inhibits bradykinin activity and stabilizes lysosomal membranes, offering effective analgesia [3]. Among the commonly used NSAIDs, ketoprofen has a low molecular weight, providing it with the highest cutaneous permeability [5], leading to better analgesia. Ketoprofen inhibits COX-1, which can cause severe gastrointestinal effects when taken orally [6]. However, when applied transdermally, the ketoprofen patch provides similar regional tissue concentrations of the active compound, despite having plasma concentrations 17 times lower [7], thereby reducing the risk of systemic complications [8]. A study by Rigourd et al. concluded that ketoprofen cannot cross the blood-milk barrier due to its hydrophilic characteristics, making it safe for breastfeeding mothers [9]. Fentanyl, a potent narcotic analgesic, has a low molecular weight and high lipophilicity, allowing for high penetration rates in the form of a transdermal patch [3]. While fentanyl patches provide strong analgesia for systemic pain, they must be administered cautiously due to potential adverse effects such as sedation, nausea, and vomiting [10].

Studies have shown that ketoprofen and fentanyl patches effectively relieve postoperative pain. Vasava and Patel found that transdermal ketoprofen was both effective and safe for acute postoperative pain after laparoscopic abdominal surgery [11], while Adelshoukry and Abddelkway observed that a 30-mg ketoprofen patch reduced the incidence of short-term post-spinal back pain [12], and Todorovic et al. concluded that a 50-mcg/h fentanyl patch provided reliable postoperative pain relief after cesarean sections with minimal side effects [13], though lower doses (12 mcg/h) were less effective in reducing the need for rescue analgesics [14]. As the ketoprofen patch is a recent development, there is limited research on its use as an analgesic. The primary objective of this research is to compare the efficacy of a 30-mg transdermal ketoprofen patch vs. a 25-mcg transdermal fentanyl patch for postoperative analgesia in women undergoing LSCS.

## Materials and methods

This prospective randomized double-blind study was conducted at the Department of Anaesthesiology, Dr. D. Y. Patil Medical Hospital and Research Center, Pimpri, Pune, from March 2024 to July 2024, after obtaining ethics committee approval. Written informed consent was obtained from all patients who agreed to participate. Double blinding was maintained throughout the study, with sealed opaque envelopes used to conceal group allocation. Data collection and analysis were conducted by an independent anesthesiologist who was not involved in the drug administration process.

The sample size was calculated based on the standard deviation (SD) of the sedation score at 20 hours, as reported in the study by Hazarika and Sarkar [15]. The SD was 0.58 for the transdermal ketoprofen patch and 0.48 for the transdermal fentanyl patch. Using a power of 95% and a significance level of 5%, the minimum sample size was calculated to be 68, rounded up to 70 (35 in each group) using the WinPepi version 11.3 (J.H. Abramson, Brixton Health, London, UK).

Patients aged 18 to 40 years, scheduled for cesarean sections, with a BMI between 20 and 25 kg/m², and who provided informed consent were included in the study. Patients with a history of NSAID allergies, opioid sensitivity, peptic ulcers, or any systemic illness such as asthma, epilepsy, chronic obstructive pulmonary disease, liver disease, cardiac disease, renal disease, coagulopathy, or contraindications to spinal anesthesia were excluded from the study.

A pilot study was conducted with 10 patients who were not included in the main study. The pilot study employed the Ramsay Sedation Scale to assess patient sedation levels among groups. However, the Ramsay Sedation Scale has limitations, particularly in its lack of detailed differentiation for agitation or restlessness, and it may not fully capture a patient’s responsiveness, especially in cases of fluctuating sedation or agitation states. Consequently, we opted to use an alternative scale in our study to address these limitations. The Richmond Agitation-Sedation Scale (RASS), commonly used in ICU settings where patients may experience varying degrees of agitation and deep sedation, was deemed more appropriate. Findings from our pilot study demonstrated that the RASS was superior to the Ramsay Sedation Scale for our specific needs. RASS allowed for a more comprehensive and nuanced assessment of sedation and agitation levels, providing better differentiation that was particularly relevant to our study population, as shown in Table 1 [16].

**Table 1:** Richmond Agitation-Sedation Scale (RASS)

RASS score	Description	Characteristics
4	Combative	Overtly combative, violent, immediate danger to staff
3	Very agitated	Pulls or removes tubes or catheters; aggressive
2	Agitated	Frequent non-purposeful movements, fights ventilator
1	Restless	Anxious but movements not aggressive or vigorous
0	Alert and calm	Alert and calm
-1	Drowsy	Sustained awakening to voice (≥10 seconds)
-2	Light sedation	Briefly awakens with eye contact to voice (<10 seconds)
-3	Moderate sedation	Movement or eye-opening to voice but no eye contact
-4	Deep sedation	No response to voice but movement or eye-opening to physical stimuli
-5	Cannot be aroused	No response to voice or physical stimulation

**Figure 1:**
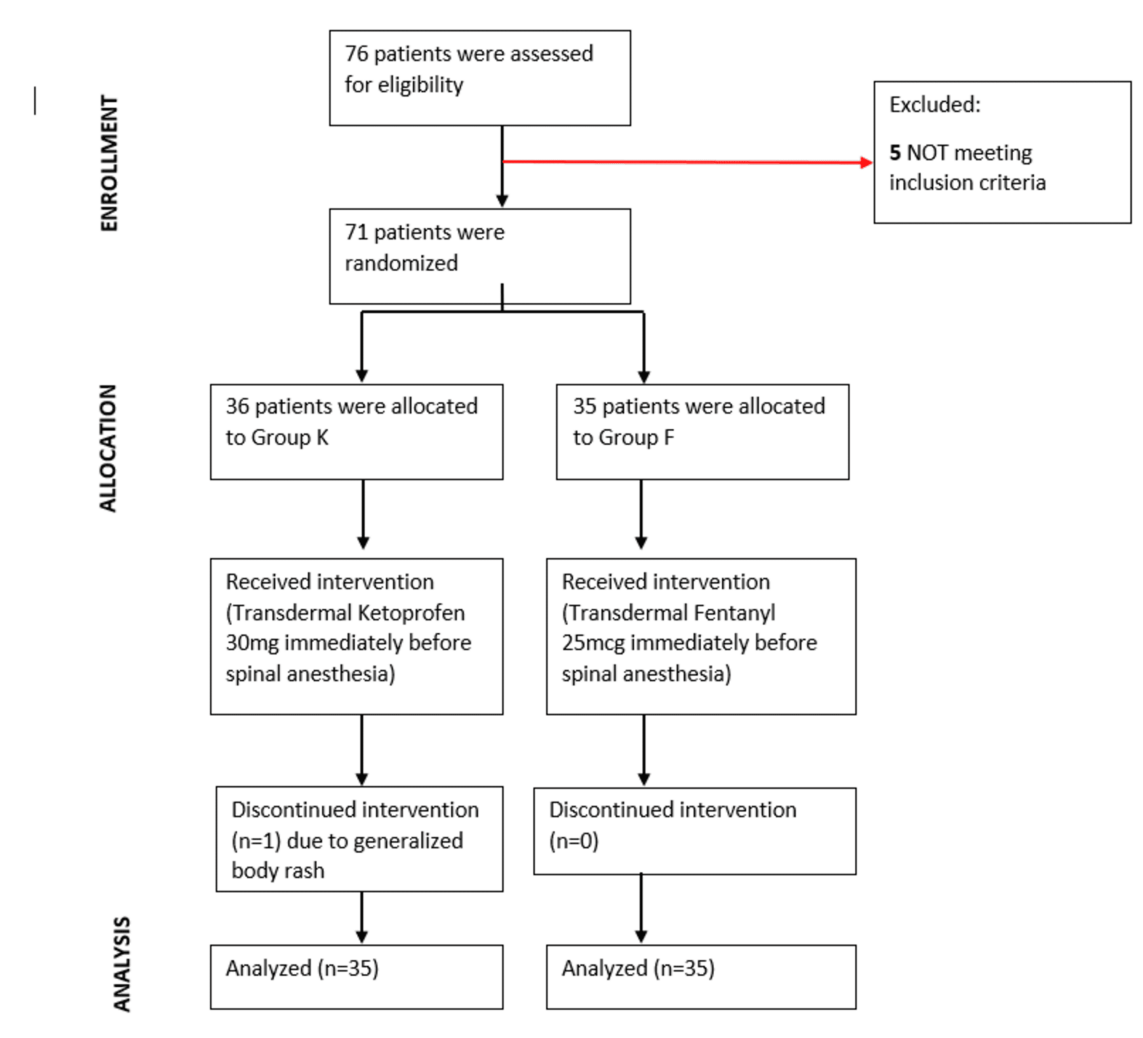
CONSORT flow chart

Figure 1 shows the CONSORT flow chart.

Seventy-one patients scheduled for LSCS were divided into two groups: Group K (n = 36), which received a ketoprofen patch (30 mg), and Group F (n = 35), which received a fentanyl patch (25 mcg). The selection was performed using a computer-generated random number table. The procedure was conducted under the supervision of an experienced anesthesiologist responsible for administering anesthesia and patient care. Only patients who met the inclusion and exclusion criteria were enrolled in the study.

After obtaining consent, a thorough pre-anesthesia checkup was performed, and all baseline parameters were recorded. Premedication included IV Ringer’s lactate (500 mL), pantoprazole injection (40 mg), and ondansetron injection (4 mg). Spinal anesthesia was administered under aseptic conditions at the L3-L4 level using a 26-G Quincke spinal needle (BD, Wokingham, UK) with 2 mL of 0.5% bupivacaine heavy. Once the patients were positioned supine, the transdermal patch was applied to the lower anterolateral chest wall immediately after the spinal anesthesia. Surgery commenced after achieving adequate sensory blockade (T6 level), and an oxytocin injection (10 IU) was administered following the delivery of the baby.

**Figure 2:**
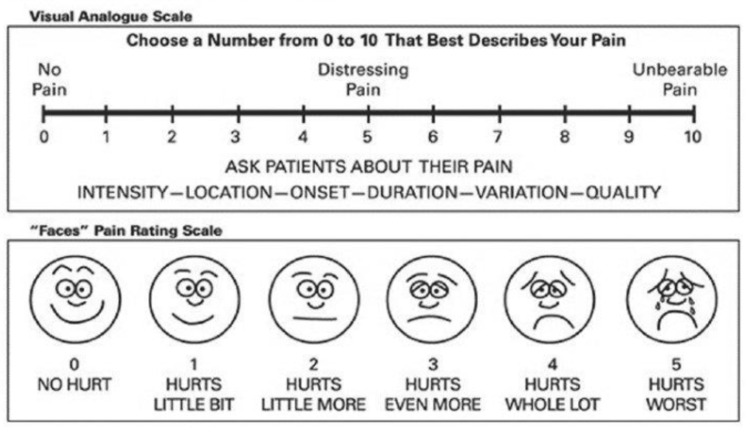
Visual Analogue Scale Reproduced from reference [17] under a Creative Common License.

Postoperative vital parameters, including heart rate, systolic blood pressure (SBP), diastolic blood pressure (DBP), mean arterial pressure (MAP), and pain scores using the Visual Analogue Scale (VAS) (Figure 2), were recorded at two-, four-, eight-, 12-, 16-, 20-, and 24-hour intervals. A VAS score of 4 or higher was treated with paracetamol injection 1 g IV. Sedation levels were assessed using the RASS at the same intervals. Any side effects such as nausea, vomiting, constipation, erythema, or indigestion were documented over the 24-hour postoperative period. One patient from Group K developed erythema and itching and was subsequently discontinued from the study.

Categorical variables were presented as numbers and percentages, while quantitative data were expressed as mean ± SD. The normality of the data was assessed using the Shapiro-Wilk test. Quantitative variables were compared using the independent t-test. For qualitative variables, the chi-square test was employed. If any cell had an expected value of less than 5, the Fisher's exact test was used instead. The Mann-Whitney U (U) test was applied for continuous data analysis. Data entry was done using Microsoft Excel (Microsoft Corp., Redmond, WA, United States), and the final analysis was performed with the IBM SPSS Statistics for Windows, Version 25.0 (Released 2017; IBM Corp., Armonk, NY, United States). A p-value of less than 0.05 was considered statistically significant.

## Results

Compared to Group F, Group K had a comparable distribution of age: 18-25 years, 20 (57.14%) in Group K vs. 22 (56.67%) in Group F and 26-35 years, 15 (42.85%) vs. 13 (43.33%) (p = 0.793). The mean ± SD of age was 24.13 ± 4.26 years in Group K and 24.3 ± 3.86 years in Group F, with no significant difference between them (p = 0.874), as shown in Table 2.

**Table 2:** Comparison of demographic details SD: standard deviation. The comparison between categorical variables (age groups) was done using the chi-square test. The comparison between means was done using the independent t-test. A p-value of less than 0.05 was considered statistically significant.

Age	Group K (n = 35)	Group F (n = 35)	Total	p-value
18-25 years	20 (57.14%)	22 (62.85%)	42 (60%)	0.625
26-35 years	15 (42.85%)	13 (37.14%)	28 (40%)	
Total	35 (100%)	35 (100%)	70 (100%)	
Mean ± SD	24.13 ± 4.26	24.3 ± 3.86	24.22 ± 4.03	0.874
Range	18-33	18-32	18-33	

No significant difference was seen in the anthropometric parameters between Group K and Group F for height (cm; p = 0.609), weight (kg; p = 0.429), and body mass index (BMI, kg/m²; p = 0.554). The mean ± SD for height, weight, and BMI in Group K were 163.03 ± 4.38 cm, 62.67 ± 4.84 kg, and 23.56 ± 1.35 kg/m², respectively, compared to 162.5 ± 3.62 cm, 61.67 ± 4.89 kg, and 23.34 ± 1.52 kg/m² in Group F, with no significant differences between the groups, as shown in Table 3.

**Table 3:** Comparison of anthropometric details SD: standard deviation. The independent t-test was used for the comparison of quantitative variables between the two groups. A p-value of less than 0.05 was considered statistically significant.

Anthropometric parameters	Group K (n = 35)	Group F (n = 35)	Total	p-value
Height (cm)				
Mean ± SD	163.03 ± 4.38	162.5 ± 3.62	162.77 ± 3.99	0.609
Range	153-169	156-168	153-169	
Weight (kg)				
Mean ± SD	62.67 ± 4.84	61.67 ± 4.89	62.17 ± 4.85	0.429
Range	50-70	50-70	50-70	
Body mass index (kg/m²)				
Mean ± SD	23.56 ± 1.35	23.34 ± 1.52	23.45 ± 1.43	0.554
Range	20.2-24.92	20.2-24.98	20.2-24.98	

Table 4 compares the hemodynamic parameters (pulse rate, SBP, DBP, and MAP]) between the two groups, Group K and Group F, over a 24-hour period. The data show the mean values and SDs at different time points (baseline to 24 hours), with p-values assessing the within-group significance over time. Both groups exhibit stable parameters without significant changes across the time intervals, except for DBP in Group F, which shows a statistically significant change (p = 0.029) at 24 hours. No other major fluctuations are observed in the other parameters.

**Table 4:** Comparison of hemodynamic parameters among groups SD: standard of deviation, SBP: systolic blood pressure, DBP: diastolic blood pressure, MAP: mean arterial pressure. p-value is analyzed using the independent t-test. p < 0.05 is highly statistically significant.

Parameters	Group	Baseline	Two hours	Four hours	Eight hours	12 hours	16 hours	20 hours	24 hours
Pulse rate (mean ± SD)	Group K	85.67 ± 11.91	79.4 ± 12.56	85.23 ± 12.29	86.73 ± 11.3	86.93 ± 12.95	86.47 ± 12.03	85.53 ± 10.73	85.27 ± 9.5
	Group F	88.7 ± 10.83	82.6 ± 11.3	83.67 ± 10.34	88.4 ± 9.06	88.37 ± 10.9	89.67 ± 9.49	88.77 ± 8.48	85.53 ± 10.08
	p-value (within group)	0.306	0.304	0.595	0.531	0.645	0.257	0.2	0.916
SBP (mean ± SD)	Group K	117.23 ± 10.17	113.33 ± 13.36	113.47 ± 8.5	114.93 ± 10.05	114.77 ± 9.82	116.33 ± 9.88	117.3 ± 7.94	116.53 ± 9.45
	Group F	119.7 ± 11.15	112.63 ± 11.38	113.83 ± 9.07	114.5 ± 8.35	117.2 ± 9	116.67 ± 9.44	115.43 ± 10.77	114.4 ± 6.79
	p-value (within group)	0.374	0.828	0.872	0.857	0.321	0.894	0.448	0.319
DBP (mean ± SD)	Group K	78.7 ± 7.03	74.37 ± 7.42	74.9 ± 11.01	75.53 ± 7.7	74.57 ± 8.97	76.73 ± 7	78.6 ± 6.87	78.03 ± 5.86
	Group F	79.47 ± 10.85	74.87 ± 10.89	73.47 ± 11.61	78.23 ± 8.38	74.63 ± 9.58	74.4 ± 9.65	75.77 ± 8.17	73.6 ± 9.03
	p-value (within group)	0.747	0.836	0.626	0.199	0.978	0.288	0.151	0.029
MAP (mean ± SD)	Group K	91.53 ± 6.97	87.36 ± 8.34	100.43 ± 6.98	87.76 ± 8.99	101.59 ± 8.93	88.67 ± 7.41	101.69 ± 7.75	87.97 ± 7.71
	Group F	92.77 ± 9.72	87.46 ± 9.36	100.84 ± 6.37	86.92 ± 8.76	100.82 ± 7.27	90.32 ± 6.97	104.21 ± 7.01	88.82 ± 7.95
	p-value (within group)	0.574	0.965	0.813	0.718	0.717	0.376	0.191	0.674

**Figure 3:**
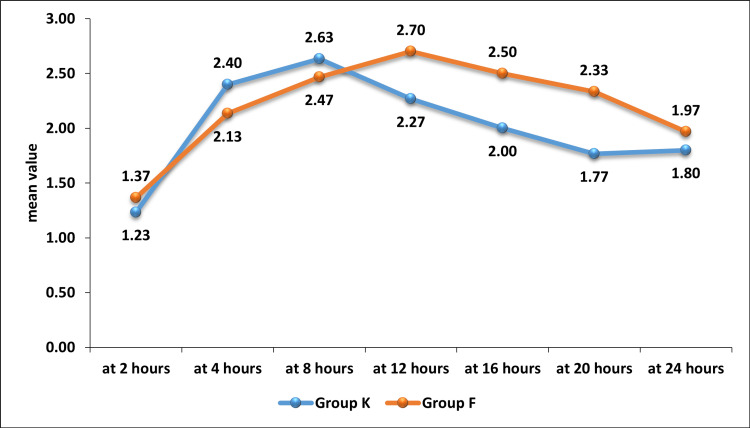
Comparison of the trend of VAS scores at different time intervals between Group K and Group F

Significant differences were seen in the VAS scores at 16 and 20 hours between Group K and Group F. The mean ± SD of the VAS scores at 16 and 20 hours in Group K were 2 ± 0.87 and 1.77 ± 0.86, respectively, which were significantly lower compared to Group F (2.5 ± 0.86, p = 0.029, and 2.33 ± 0.8, p = 0.011, respectively), as shown in Table 5 and Figure 3.

**Table 5:** Comparison of VAS scores between Group K and Group F VAS: Visual Analogue Scale. The independent t-test was used for the comparison of quantitative variables (VAS score) between the two groups. A p-value of less than 0.05 was considered statistically significant.

VAS score	Group K (n = 35)	Group F (n = 35)	Total	p-value
At two hours				
Mean ± SD	1.23 ± 0.43	1.37 ± 0.56	1.3 ± 0.5	0.303
Range	1-2	0-2	0-2	
At four hours				
Mean ± SD	2.4 ± 1.07	2.13 ± 0.57	2.27 ± 0.86	0.235
Range	1-4	1-3	1-4	
At eight hours				
Mean ± SD	2.63 ± 0.96	2.47 ± 0.97	2.55 ± 0.96	0.508
Range	1-4	1-4	1-4	
At 12 hours				
Mean ± SD	2.27 ± 0.87	2.7 ± 0.95	2.48 ± 0.93	0.071
Range	1-4	1-4	1-4	
At 16 hours				
Mean ± SD	2 ± 0.87	2.5 ± 0.86	2.25 ± 0.89	0.029
Range	1-5	1-4	1-5	
At 20 hours				
Mean ± SD	1.77 ± 0.86	2.33 ± 0.8	2.05 ± 0.87	0.011
Range	1-4	1-4	1-4	
At 24 hours				
Mean ± SD	1.8 ± 0.92	1.97 ± 0.76	1.88 ± 0.85	0.45
Range	1-4	1-3	1-4	

At the 20-hour mark, significantly more patients in Group K were alert and calm compared to those in Group F, with 94.29% (33 patients) in Group K vs. 74.29% (26 patients) in Group F. Group K also had fewer drowsy or restless patients than Group F: no patients (0%) in Group K were drowsy, while 14.29% (5 patients) in Group F were; 5.71% (2 patients) in Group K were restless, compared to 11.43% (4 patients) in Group F (p = 0.044).

At 24 hours, this trend continued. Group K had a significantly lower proportion of drowsy patients at 0% (no patients) vs. 37.14% (13 patients) in Group F. Conversely, a higher proportion of Group K patients were alert and calm at 97.14% (34 patients) compared to 54.29% (19 patients) in Group F. Additionally, Group F had more restless patients at 8.57% (three patients) than Group K at 2.86% (one patient) (p < 0.0001), as shown in Table 6.

**Table 6:** Comparison of the Richmond Agitation-Sedation Scale among groups The chi-square test was used to compare the categorical data (Richmond Agitation-Sedation Scale between Groups K and F). A p-value of less than 0.05 was considered statistically significant.

Richmond Agitation-Sedation Scale	Group K (n = 35)	Group F (n = 35)	Total	p-value
At two hours				
Drowsy	7 (20%)	7 (20%)	14 (20%)	0.509^*^
Alert and calm	27 (77.14%)	24 (68.57%)	51 (72.86%)	
Restless	1 (2.86%)	4 (11.43%)	5 (7.14%)	
At four hours				
Drowsy	0 (0%)	3 (8.57%)	3 (4.29%)	0.157^*^
Alert and calm	28 (80%)	28 (80%)	56 (80%)	
Restless	7 (20%)	4 (11.43%)	11 (15.71%)	
At eight hours				
Drowsy	0 (0%)	0 (0%)	0 (0%)	1.0^*^
Alert and calm	28 (80%)	29 (82.86%)	57 (81.43%)	
Restless	7 (20%)	6 (17.14%)	13 (18.57%)	
At 12 hours				
Drowsy	0 (0%)	0 (0%)	0 (0%)	0.218^*^
Alert and calm	31 (88.57%)	26 (74.29%)	57 (81.43%)	
Restless	4 (11.43%)	9 (25.71%)	13 (18.57%)	
At 16 hours				
Drowsy	0 (0%)	1 (2.86%)	1 (1.43%)	0.259^*^
Alert and calm	33 (94.29%)	29 (82.86%)	62 (88.57%)	
Restless	2 (5.71%)	5 (14.29%)	7 (10%)	
At 20 hours				
Drowsy	0 (0%)	5 (14.29%)	5 (7.14%)	0.044^*^
Alert and calm	33 (94.29%)	26 (74.29%)	59 (84.29%)	
Restless	2 (5.71%)	4 (11.43%)	6 (8.57%)	
At 24 hours				
Drowsy	0 (0%)	13 (37.14%)	13 (18.57%)	<0.0001^*^
Alert and calm	34 (97.14%)	19 (54.29%)	53 (75.71%)	
Restless	1 (2.86%)	3 (8.57%)	4 (5.71%)	

Compared to Group K, Group F had a comparable distribution of the requirement for rescue analgesia. The proportions were as follows: seven patients (20%) in Group K vs. 12 patients (34.29%) in Group F required rescue analgesia, and 28 patients (80%) in Group K vs. 23 patients (65.71%) in Group F did not require rescue analgesia (p = 0.282), as shown in Table 7.

**Table 7:** Comparison of the number of patients required rescue analgesia among Group K and Group F The chi-square test was used to compare the categorical data (requirement of rescue analgesia between Groups K and F). A p-value of less than 0.05 was considered statistically significant.

Requirement of rescue analgesia	Group K (n = 35)	Group F (n = 35)	Total	p-value
Yes	7 (20%)	12 (34.29%)	19 (27.14%)	0.282
No	28 (80%)	23 (65.71%)	51 (72.86%)	
Total	35 (100%)	35 (100%)	70 (100%)	

Table 8 compares the number of patients in Group K and Group F who required rescue analgesia and the total dose of paracetamol needed. Group K had seven patients needing 8 g of paracetamol, while Group F had 12 patients requiring 14 g. This suggests that Group F required more rescue analgesia and a higher total dose of paracetamol compared to Group K.

**Table 8:** Comparison of the total dose of rescue analgesia required among Group K and Group F The chi-square test was used to compare the number of patients requiring rescue analgesia between the two groups. The independent t-test was used to compare the total dose of paracetamol required between the two groups. A p-value of less than 0.05 was considered statistically significant.

Parameters	Group K	Group F	p-value
Number of patients requiring rescue analgesia dose	7	12	0.282
Total dose of paracetamol required (g)	8	14	0.026

Table 9 compares the mean values of a variable between Group K and Group F. Group K has a mean of 0.22 (SD 0.4902), while Group F has a mean of 0.4 (SD 0.5530). The U test result (U = 504, p = 0.204) indicates that there is no statistically significant difference between the two groups (p > 0.05).

**Table 9:** Statistical analysis for rescue analgesia required among groups The Mann-Whitney U test (U) was applied to compare the two groups since the data did not follow a normal distribution. A p-value of less than 0.05 was considered statistically significant.

Group	Mean (SD)	Significance
Group K	0.22 (0.4902)	U= 504, p= 0.204
Group F	0.4 (0.5530)	

Compared to Group F, Group K had comparable distribution of various complications. The proportions were as follows: no complications in 29 (82.86%) in Group K vs. 22 (62.86%) in Group F (p = 0.108). Specific complications included constipation, 0 (0%) vs. 5 (14.29%), with a p-value of 0.112; indigestion, 1 (2.86%) vs. 0 (0%), with a p-value of 1; itching, 3 (8.57%) vs. 2 (5.71%), with p = 0.612; nausea, 1 (2.86%) vs. 5 (14.29%), with p = 0.353; rash, 1 (2.86%) vs. 0 (0%), with p = 1; and vomiting, 0 (0%) vs. 1 (2.86%), with p = 1, as shown in Table 10.

**Table 10:** Comparison of complications among groups The chi-square test was applied for statistical analysis. The Fisher's exact test was used when any cell had an expected value of less than 5. A p-value of less than 0.05 was considered statistically significant.

Various complications	Group K (n = 35)	Group F (n = 35)	Total	p-value
Nil	29 (82.86%)	22 (62.86%)	51 (72.86%)	0.108
Constipation	0 (0%)	5 (14.29%)	5 (7.14%)	0.112
Indigestion	1 (2.86%)	0 (0%)	1 (1.43%)	1
Itching	3 (8.57%)	2 (5.71%)	5 (7.14%)	0.612
Nausea	1 (2.86%)	5 (14.29%)	6 (8.57%)	0.353
Rash	1 (2.86%)	0 (0%)	1 (1.43%)	1
Vomiting	0 (0%)	1 (2.86%)	1 (1.43%)	1

## Discussion

The key findings of our study indicate that the 30-mg ketoprofen patch provides effective postoperative pain relief with fewer side effects and lower sedation levels compared to the 25-mcg/h fentanyl patch in patients undergoing cesarean sections. The VAS scores demonstrated that Group K achieved significantly better pain control at 16 hours (VAS score 2 ± 0.87 vs. 2.5 ± 0.86, p = 0.029) and at 20 hours (VAS score 1.77 ± 0.86 vs. 2.33 ± 0.8, p = 0.011) compared to Group F. Furthermore, a lower proportion of patients in Group K required rescue analgesia compared to Group F (20% vs. 34.29%, p = 0.282), though this difference was not statistically significant.

Postoperative pain management after cesarean sections poses a challenge, particularly when the mother needs to assist with breastfeeding and baby care. McCaffrey and Ferrell [18] reported that over 50% of surgical patients experience inadequate pain relief, which can result in negative psychological and physiological consequences. The ketoprofen patch, a recent introduction, has been studied for its safety during breastfeeding. Research by Rigourd et al. concluded that ketoprofen, due to its lipophilic properties, does not cross the blood-milk barrier [9]. This formed the rationale for using the ketoprofen patch as an analgesic in our study after cesarean sections, given the limited research on its efficacy in this context.

Since higher doses of transdermal fentanyl patches (50 mcg/h and 75 mcg/h) can cause sedation that may interfere with breastfeeding, we opted for the lowest effective dose (25 mcg/h) for comparison with the 30-mg ketoprofen patch. Rallabhandi et al. [19] demonstrated that a 25-mcg/h transdermal fentanyl patch provides effective, noninvasive postoperative pain relief. At two hours post-surgery, we observed low VAS scores, likely due to the residual effect of spinal anesthesia: Group K recorded a VAS score of 1.2 ± 0.43 and Group F had a score of 1.37 ± 0.56 (p = 0.303). Between four and 24 hours, the VAS scores ranged from 2.4 to 1.8 in Group K and from 2.13 to 1.97 in Group F. Notably, Group K showed significantly better pain relief at 16 hours (VAS score 2 ± 0.87 vs. 2.5 ± 0.86, p = 0.029) and 20 hours (VAS score 1.77 ± 0.86 vs. 2.33 ± 0.8, p = 0.011).

Unlike our findings, Hazarika and Sarkar [15] reported peak analgesic effects in the fentanyl group at 12 hours postoperatively. The discrepancy may arise from their application of the fentanyl patch four hours before surgery, whereas we applied the patches immediately after spinal anesthesia. Further research with larger sample sizes is necessary to confirm the analgesic efficacy of both treatments. Rallabhandi et al. [19] found peak fentanyl analgesic effects at six hours postoperatively, which differs from our results, likely due to their application of the patch 10 hours prior to surgery. Additionally, a study by Bharath et al. [20] demonstrated that fentanyl was more effective than ketoprofen after mandibular third molar surgery, possibly due to the low dose of transdermal ketoprofen (20 mg) they used. They also employed a different pain assessment scale, the Verbal Pain Intensity and Pain Relief chart (0-4 scale), whereas we used the VAS (0-10 scale).

In our study, seven (20%) of the patients in Group K (seven out of 35) required rescue analgesia, compared to 12 (34.29%) in Group F (12 out of 35), although the difference was not statistically significant (p = 0.282). Group F patients required a total of 14 g of paracetamol, while those in Group K required 8 g, with no significant difference in the total dose of rescue analgesia (p = 0.204). In terms of sedation scores, both groups had similar distributions up to 16 hours. At 20 hours, however, Group F had significantly more drowsy patients (5 (14.29%) vs. 0 (0%), p = 0.044), while Group K had a higher proportion of patients who were alert and calm (33 (94.29%) vs. 26 (74.29%)). At 24 hours, 13 (37.14%) of the patients in Group F were drowsy, compared to none in Group K (p < 0.0001), while Group K had a significantly higher number of alert and calm patients (34 (97.14%) vs. 19 (54.29%)).

Sevarino et al. state that the incidence of respiratory depression, sedation, and nausea or vomiting was high when transdermal fentanyl 75 mcg/h was used for postoperative pain management [21]. Certain physiological changes during pregnancy, such as increased progesterone levels and altered volume of distribution, affect drug pharmacokinetics, reducing opioid requirements. Rallabhandi et al. reported that transdermal fentanyl 25 mcg/h provides an effective and noninvasive method of postoperative pain relief after major abdominal surgery [19]. To avoid respiratory depression and other side effects, this study chose the minimum effective dose, that is, transdermal fentanyl 25 mcg/h.

No serious systemic adverse effects were observed in either group. One patient in the ketoprofen group experienced indigestion, and three patients experienced itching, compared to two in the fentanyl group. Nausea was more common in Group F (five patients) than in Group K (one patient). One patient in Group K developed a rash, leading to the removal of the ketoprofen patch, while five patients in Group F experienced constipation. One patient in Group F also had vomiting. As postoperative complications depend on multiple factors, such as anxiety, food intake, medication use, and psychological factors, larger studies are necessary to better understand these outcomes.

The limitations of this study include the need for more research on ketoprofen's efficacy in a larger sample size and across both genders, as pain perception can vary between men and women. Additionally, while we assessed analgesic efficacy over 24 hours, further studies are required to evaluate longer-term efficacy. Future research comparing different doses of ketoprofen and fentanyl patches would also help identify equi-analgesic doses at all time points.

## Conclusions

In conclusion, both transdermal ketoprofen (30 mg) and fentanyl (25 mcg) patches provided effective analgesia for cesarean section patients, with ketoprofen offering superior pain relief beyond 12 hours and fewer sedation-related effects at 16 and 20 hours postoperatively. Ketoprofen patients were more alert, supporting better recovery and enabling engagement in essential postpartum activities like breastfeeding. Further research is recommended to explore the long-term benefits of transdermal analgesics for postoperative care.
